# First Qualification Study of Serum Biomarkers as Indicators of Total Body Burden of Osteoarthritis

**DOI:** 10.1371/journal.pone.0009739

**Published:** 2010-03-17

**Authors:** Virginia B. Kraus, Thomas B. Kepler, Thomas Stabler, Jordan Renner, Joanne Jordan

**Affiliations:** 1 Department of Medicine, Duke University Medical Center, Durham, North Carolina, United States of America; 2 Department of Biostatistics and Bioinformatics and Department of Immunology, Duke University Medical Center, Durham, North Carolina, United States of America; 3 Thurston Arthritis Research Center, University of North Carolina at Chapel Hill, Chapel Hill, North Carolina, United States of America; University of Otago, New Zealand

## Abstract

**Background:**

Osteoarthritis (OA) is a debilitating chronic multijoint disease of global proportions. OA presence and severity is usually documented by x-ray imaging but whole body imaging is impractical due to radiation exposure, time and cost. Systemic (serum or urine) biomarkers offer a potential alternative method of quantifying total body burden of disease but no OA-related biomarker has ever been stringently qualified to determine the feasibility of this approach. The goal of this study was to evaluate the ability of three OA-related biomarkers to predict various forms or subspecies of OA and total body burden of disease.

**Methodology/Principal Findings:**

Female participants (461) with clinical hand OA underwent radiography of hands, hips, knees and lumbar spine; x-rays were comprehensively scored for OA features of osteophyte and joint space narrowing. Three OA-related biomarkers, serum hyaluronan (*s*HA), cartilage oligomeric matrix protein (*s*COMP), and urinary C-telopeptide of type II collagen (*uCTX2*), were measured by ELISA. *s*HA, *s*COMP and *u*CTX2 correlated positively with total osteophyte burden in models accounting for demographics (age, weight, height): R^2^ = 0.60, R^2^ = 0.47, R^2^ = 0.51 (all p<10^−6^); *s*COMP correlated negatively with total joint space narrowing burden: R^2^ = 0.69 (p<10^−6^). Biomarkers and demographics predicted 35–38% of variance in total burden of OA (total joint space narrowing or osteophyte). Joint size did not determine the contribution to the systemic biomarker concentration. Biomarker correlation with disease in the lumbar spine resembled that in the rest of the skeleton.

**Conclusions/Significance:**

We have suspected that the correlation of systemic biomarkers with disease has been hampered by the inability to fully phenotype the burden of OA in a patient. These results confirm the hypothesis, revealed upon adequate patient phenotyping, that systemic joint tissue concentrations of several biomarkers can be quantitative indicators of specific subspecies of OA and of total body burden of disease.

## Introduction

Osteoarthritis (OA) is a debilitating chronic multijoint disease with major global impact [1]. OA presence and severity is usually documented by x-ray imaging but whole body imaging is impractical due to radiation exposure, time and cost. Systemic (serum or urine) biomarkers offer a potential alternative method of quantifying total body burden of disease but no OA-related biomarker has ever been stringently validated to determine the feasibility of this approach. Systemic biomarkers in OA are usually only assessed relative to a particular joint site under study. The advent of disease status indicators in the serum or urine would provide badly needed objective means of evaluating and monitoring the disease, could provide quantitative traits for genetic studies, and could contribute to identifying individuals at greatest risk of progression, and therefore, in greatest need of prevention strategies.

The inference of the relevant relationships between serum or urine biomarker concentrations and disease of specific joints is complicated by the fact that many or most OA patients have disease in multiple joints. Moreover, OA is a complex disease increasingly understood to be a mixture of distinct clinical and genetic entities [2], [3], [4], [5]. OA of the knee may or may not share the same etiology as OA of the spine or hands. Biomarkers may be useful as indicators of specific subspecies of OA, or of OA in specific joints. The distinct features of radiographic OA of joint space loss and osteophyte represent different disease processes that may also differ in their associations with biomarkers. These features are highly correlated so it is necessary to adjust for this correlation in order to test the independent association of biomarkers with particular features of OA. Any study aimed at elucidating these relationships will need to collect extensive radiological data to survey all joints potentially involved in OA. Only a small fraction of the patients analyzed will have OA isolated to one set of joints [6]. To focus on this small fraction of the total data would be wasteful and unsatisfying. Instead, we have chosen to analyze the full dataset, relying on statistical analysis, model selection, and model averaging to disentangle the web of correlations among radiological disease, OA risk factors, and biomarkers.

Qualification is the evidentiary process of linking a biomarker with biological processes and clinical end points [7]. In this work, we focus on the process of biomarker qualification for structural endpoints of OA, such as osteophyte formation and radiographic joint space narrowing, as opposed to symptomatic endpoints such as joint pain. We chose to analyze three biomarkers in this study based on the strength of previous evidence showing associations with OA: serum hyaluronan (sHA), serum cartilage oligomeric matrix protein (sCOMP), and urinary C-terminal propeptide epitope of collagen II (uCTX2). Each of these biomarkers has data to support its classification [8] in at least two categories of the BIPED [9] biomarker classification scheme (Burden of disease, Investigational, Prognostic, Efficacy of intervention, and Diagnostic biomarkers). The categories to which each of these biomarkers corresponds for structural features of OA are as follows: HA–categories D, B, P, E [10], [11], [12], [13], [14], [15], [16]; COMP–categories D, B, P [16], [17], [18]; CTX2–categories D, B, P E [10], [15], [19], [20], [21], [22], [23].

In this work we describe which radiological features are associated with each systemic biomarker and the extent to which these biomarkers reflect burden of disease of the various forms and features of OA. Here we have qualified these three biomarkers as strong quantitative traits of various features of OA. Uncoupling these features or processes of OA provided insights into the origin of these biomarkers. We observed that the canonical radiographic features of OA, namely osteophyte and joint space narrowing, correlated with one another, often in a complex manner with regard to their effects on a biomarker. We further observed that small joints did not necessarily contribute in a small way to serum biomarker concentrations. Moreover, with respect to the impact on biomarkers, the lumbar spine radiographic features behaved similarly to radiographic features in other joints, arguing for a similar disease process ongoing in the lumbar spine as in the rest of the skeleton. Finally, systemic biomarkers were more powerful predictors of multijoint disease than of disease in any single joint when added to a statistical model containing risk factor data (age, height, and weight). A notable exception was the knee in which biomarkers doubled the power of these factors alone for predicting radiographic features of OA.

## Results

### Participant characteristics

A total of 461 female participants were studied. Their mean (SD) age was 67 (10) years old. Their mean (SD) weight, height and body mass index were 74 (17) kg, 161 (7) cm, and 28 (6)_(kg/m^2^), respectively. Just over two-thirds of the participants were evaluated through the Duke site (n = 322) and the remainder at the UNC site (n = 139).

### Correlations and bilateral symmetry among radiographic features of OA in the various joint systems

There were seven distinct joint systems considered: distal (DIP) and proximal (PIP) interphalangeal finger joints, metacarpophalangeal (MCP or knuckle) hand joints, carpometacarpal (CMC or base of thumb) joints, knees, hips and lumbar spine. Figure S1A-B shows the age-related increased prevalence of affected joint systems based on any osteophyte or any joint space narrowing scores; Figure S1C shows the numbers of subjects by decade of age with with the OA affected joint system based on the Kellgren Lawrence grade >1 grade (Fig. S1C). age-related increased prevalence of affected joint systems based on the traditional Kellgren Lawrence (KL) grading system [24] (quantified in Table S1).

**Figure 1:**
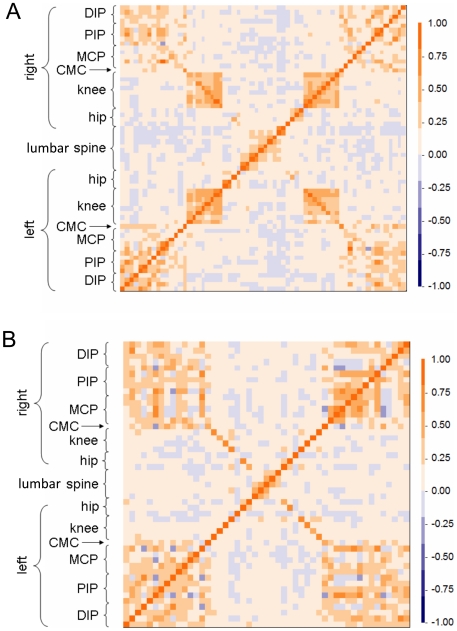
Correlation matrix of radiographic features of osteoarthritis. The correlation for left and right joints are shown for A) osteophyte score in each pair of joint faces, B) joint space narrowing score for each pair of faces. Each cell indicates the correlation between two joint faces as indicated in the color legend. The correlation matrix is symmetric by definition across the main diagonal. Left and right sides of the body are depicted from bottom to top on the y-axis and from left to right on the x-axis.

Figure 1 shows the correlation matrix among joints affected by osteophyte (OST), and by joint space narrowing (JSN), a surrogate for cartilage loss. The tapestries are complex, reflecting the inherent complexity of OA, but the departures from randomness apparent in their patchiness and symmetries provide important insights into the relationships among the manifestations of disease in the various joints. Two interesting features are discernible in this figure. First, joint faces (medial, lateral, superior and inferior joint margins) within the same joint tended all to be affected by OST, as indicated by the appearance of square patches in the resulting tapestry of this matrix (Figure 1A), particularly in the knees, hands and lumbar spine. Such correlation within joints was less evident for JSN (Figure 1B) but still apparent in the hands, and to a lesser extent, the lumbar spine. Second, there was a high degree of bilateral symmetry in the distribution of OA in these study participants as indicated by the mirror-image symmetry of both panels of Figure 1 across the diagonal. In the JSN tapestry (Figure 1B), this bilateral symmetry extended even to the preferential correlation of the same specific joint faces in contralateral joints, as was especially apparent, for example, in the knees and hips (shown by the strong backward diagonal line). In addition, there was greater collateral involvement of neighboring joints on the right hand, based on JSN, than the left hand (top right versus bottom left corner of Figure 1B). This further highlights the striking multi-joint OA involvement of individuals recruited on the basis of hand OA alone.

For each participant, we summed the total number of joint faces with OST >0 and the total number of joints with JSN >0, designating these sums *n_OST_* and *n_JSN_*, respectively. We also summed the total number of joints with OA based on KL scoring (KL>1), designating this sum *n_KL_*. Among participants, *n_OST_* and *n_JSN_* had a correlation coefficient of r = 0.66, which was very highly significant (p<10^−6^).

### Associations of biomarkers and radiological features of OA

The OA-related biomarkers, serum hyaluronan (*s*HA) and urinary C-telopeptide fragment of type II collagen (*u*CTX2), were strongly positively correlated with the number of affected joint systems based on KL scoring, with their concentrations having significantly positive coefficients in a linear model with *n_KL_* as a predictor (Figure S2). Quite surprisingly, serum cartilage oligomeric matrix protein (*s*COMP) showed a statistically significant (p = 0.024) concentration decrease as *n_KL_* increased. Note that these models were fit with the following risk factors: [12], [17], [25] age, log height, log weight for *s*HA; age for *s*COMP; and for *u*CTX2 no other covariate was used. These covariates were tested based on their significance in previous work [12], [17], [25], [26], [27],and those used for these analyses were based on their significant association with OA in models that included the biomarkers.

**Figure 2:**
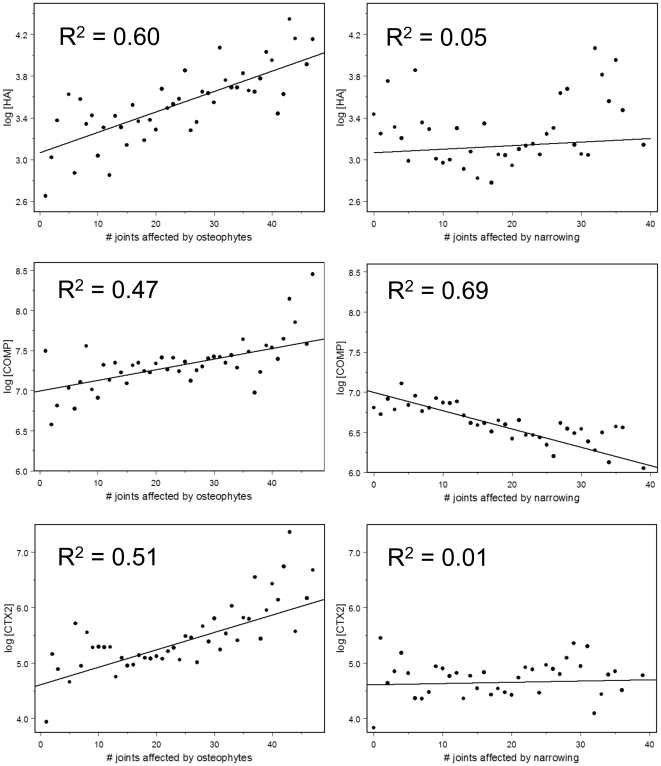
Biomarkers and total burden of OA based on radiographic features (adjusted). The data are restricted to female subjects with complete radiographic and biomarker data. The data were fit to a mixed-effects model, with family as the grouping variable for the random effect. The fixed effects are the numbers of joint faces affected by osteophytes and joint-space narrowing, respectively. Point estimates are plotted for mean serum log *s*HA (top), log *s*COMP (middle), and log *u*CTX2 (bottom) by number of joint faces affected by any osteophyte (grade >0, left panels), and number of joint faces OA affected by any joint space narrowing (grade >0, right panels). Joint faces are counted without regard to which joint they belong. JSN point estimates account for OST and OST point estimates account for JSN. In addition, sHA was adjusted for age, log height, log weight; sCOMP was adjusted for age; and CTX2 did not require adjustment. The R^2^ values indicated give the proportion of the inter-radiographic class variation explained by the regression. The respective generalized R^2^ values for the variance explained by the radiographic features are 4.4%, 6.2%, and 10.2%. All p-values for the weighted regressions are less than 10^−6^, except for the pairing of *s*HA and JSN (p = 0.18), and *u*CTX2 and JSN (p = 0.51). The mean (standard deviation) and range of biomarker concentrations prior to logarithmic transformation were: *s*HA mean 50.7(56.5) ng/ml, range: 1.1–499 ng/ml; *s*COMP mean 1130(620) ng/ml, range: 203–4903 ng/ml; *u*CTX2 mean 372(806) ng/mmol, range: 19.6–12,171 ng/mmol.

The KL scoring system is a composite variable, consisting of some OST and some JSN criteria. To assess these features uncoupled, we evaluated the association of the biomarkers to OST and JSN in models fit with the risk factors noted above and with *n_OST_* and *n_JSN_* (Figure 2), i.e. joint faces were counted without regard to which joint they belonged to and we accounted for correlations among features. Accounting for correlation with JSN, all three biomarkers showed a positive dose-dependent relationship with total OST burden (p<10^−6^). In contrast, although *s*HA and *u*CTX2 showed a strong positive dose-dependent relationship with total JSN burden before adjustment for OST (Figure S3), there was no significant association of *s*HA or *u*CTX2 with JSN after accounting for correlation with OST (Figure 2). Surprisingly perhaps, and in contrast to *s*HA and *u*CTX2, *s*COMP showed a negative dose-dependence with total JSN-affected faces, even after accounting for OST (Figure 2). Some of the most striking associations between individual biomarkers and specific radiographic features are shown in Figure S4: namely sHA and knee OST and CMC JSN; sCOMP and DIP JSN (inverse correlation); and uCTX2 and hip OST.

**Figure 3:**
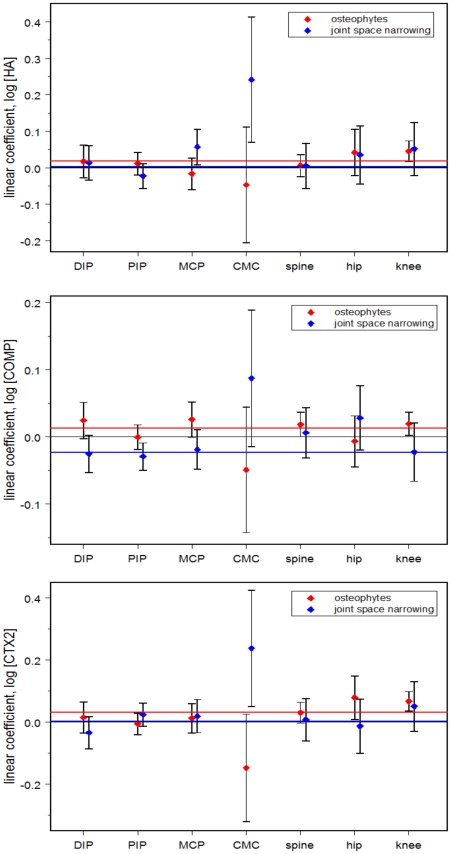
Plot of linear coefficients of biomarker concentrations and radiographic features of OA for 7 joint systems (adjusted). The linear coefficient was derived for the association of each biomarker with OST and JSN for each joint site. The mean linear coefficient from all joint sites is represented by the red line (for OST) and the blue line (for JSN) depicting the strength of the association. The coefficients are shown for serum log *s*HA (top), log *s*COMP (middle), and log *u*CTX2 (bottom) by number of joint faces affected by any osteophyte (grade >0), and number of joint faces affected based on any joint space narrowing (grade >0). All features were included in each model to assess the contribution of each feature in the presence of the others, i.e. we accounted for correlations among features. Error bars represent 95% confidence intervals.

We next computed the regression coefficients for each biomarker (dependent variable) for each joint site and radiographic feature (independent variables) in a mixed-effects model with all 14 distinct radiographic features (OST and JSN for each of 7 joint systems) included as predictors. This “saturated” model was not optimally predictive (we developed an optimized model below), but it did allow us to effectively decouple the correlations among the independent variables that would otherwise confound our estimation of the relationships between them and the biomarkers, and to evaluate the contributions of each in its natural context. In Figure 3, a visual comparison is shown of these coefficients considered jointly, through simultaneous fits to all radiographic features; Figure S5 shows the same coefficients estimated by fitting to single radiographic features individually, i.e. not accounting for correlations among features. In both cases, the horizontal reference lines are located at the common coefficient value obtained by considering all joints equivalent, ie, constraining all joints to have identical coefficients. Coefficients significantly above these lines identify features of a particular joint that contribute disproportionately (higher than expected assuming equivalence among joint systems) to the respective biomarker concentrations–while coefficients below the reference lines identify features of a particular joint that provide less than the average contribution to the respective biomarker concentration. Noteworthy findings illustrated in Figure 3 include the fact that CMC JSN contributed disproportionately (positively) to all three biomarker concentrations, while CMC OST had the opposite tendency (disproportionate negative contribution). Knee OST and knee JSN contributed greater than average levels to both *s*HA and *u*CTX2. These plots again demonstrate the positive association of *s*COMP with OST, but a negative association, with the exception of JSN of the CMC, of *s*COMP with JSN.

Due to the interrelationship of JSN and OST, (illustrated below for the CMC joint and an issue for most other joints as well), misleading conclusions can be drawn by examining given radiographic features in isolation from the rest. Figure S5 (lower panel) shows the results of such an unadjusted analysis, where OST or JSN in each joint was used as the sole predictor for *u*CTX2. There are some notable differences between these results and those shown in the *u*CTX2 panel of Figure 3 (adjusted). In both the unadjusted and adjusted analyses, uCTX2 is associated with CMC JSN. In contrast, uCTX2 was associated with CMC OST in the unadjusted analyses but when OST and JSN are accounted for in all joints, the association of uCTX2 and OST not only declined, it became nominally negative, with borderline statistical significance. Figure S6 illustrates the interaction of OST and JSN as contributors to *u*CTX2 for the CMC joint. It shows that when CMC joint faces affected by OST are measured alone they indicate the likelihood of joint faces affected by JSN, which in turn is predictive of increased levels of *u*CTX2. If both CMC JSN and OST are measured, greater OST involvement predicts a lower *u*CTX2 concentration.

### Optimal prediction of biomarker concentration

**Figure 4:**
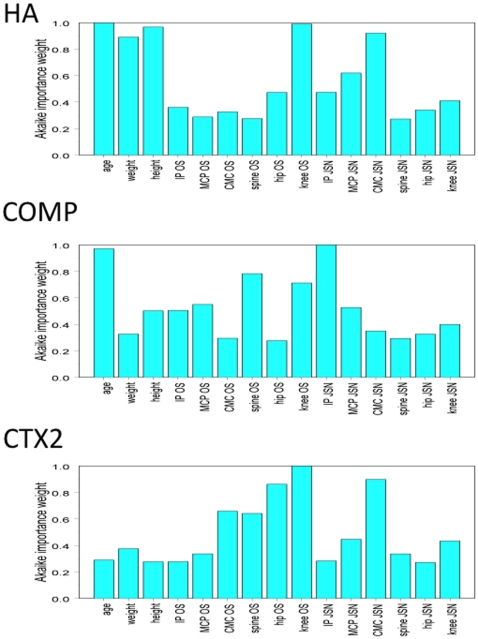
Akaike importance weights for each radiographic feature in linear models for the biomarker concentrations. All first-order linear models were fit, and the Akaike weight computed for each one. The bar height is the Akaike importance weight, the sum of the Akaike weights over all models: the greater the height, the more predictive of biomarker concentration.

Because radiographic features have the potential to confound each other, we set out to determine the best combination of features for the prediction of biomarker concentrations. We considered all linear mixed-effects models with family, age, height, and weight as well as JSN and OST of each of the seven joint systems as possible linear predictors. For some analyses, we combined PIP and DIP finger joints into a single class, interphalangeal joints (IP). Since each of these terms can be either included or excluded in any given model, there are 2^17^ = 131,072 models to evaluate, which we did exhaustively. We used the Akaike Information Criterion (AIC) [28], [29] to evaluate each model. The resulting optimal models are shown in Table 1. Notably, *u*CTX2, distinct from the other two biomarkers, had no significant relationship to age, height or weight after accounting for the radiographic features of OA. IP JSN was negatively predictive of *s*COMP concentrations. In addition, we computed the Akaike importance weights to examine the relative importance of each joint system apart from their role in the single optimal model (Figure 4). The Akaike weight for a given model is proportional to exp(-AIC/2). The weights are normalized to sum to one over all models in the class under consideration. The Akaike importance weight for a predictor is the sum of Akaike weights over all models in which that predictor is included. The Akaike importance weights (0–1) are intended to provide a measure of the importance of a predictor with respect to a whole class of models, rather than to any individual model, and are thus robust against model mis-specification [29]. Shown in Table 1 (taking into account all joints): sHA reports on MCP JSN, CMC JSN, hip OST and knee OST; sCOMP reports on IP JSN (inverse correlation), MCP OST, MCP JSN (inverse correlation), lumbar spine OST, and knee OST; and uCTX2 reports on CMC OST (inverse correlation), CMC JSN, lumbar spine OST, hip OST and knee OST.

**Table 1:** Covariates and their coefficients comprising the minimum-AIC models for predicting *s*HA, *s*COMP and *u*CTX2.

	OA Feature	*s*HA	*s*COMP	*u*CTX2
**age**		0.018 (0.005)	0.009 (0.003)	
**weight**		0.49 (0.18)		
**height**		−2.75 (0.90)		
**IP**	**OST**			
	**JSN**		−0.023 (0.005)	
**MCP**	**OST**		0.023 (0.012)	
	**JSN**	0.036 (0.022)	−0.026 (0.014)	
**CMC**	**OST**			−0.163 (0.084)
	**JSN**	0.165 (0.046)		0.270 (0.089)
**Lumbar Spine**	**OST**		0.019 (0.008)	0.029 (0.015)
	**JSN**			
**Hip**	**OST**	0.042 (0.030)		0.080 (0.034)
	**JSN**			
**Knee**	**OST**	0.050 (0.011)	0.013 (0.006)	0.083 (0.012)
	**JSN**			
	**R^2^ without radiographic data**	15.3%	0.1%	NA
	**R^2^ with radiographic data**	24.3%	8.8%	16.5%

The numbers are parameter estimates with their standard errors in parentheses.

AIC  =  Akaike's Information Criterion.

Percent biomarker variance explained (generalized R^2^) with and without radiographic data (i.e. IP MCP, CMC, spine, hip and knee data).

IP  =  interphalangeal (distal and proximal) finger joints.

MCP  =  metacarpophalangeal (knuckle) hand joints.

CMC  =  first carpometacarpal (base of thumb) joint.

*s*HA  =  serum hyaluronan.

*s*COMP  =  serum cartilage oligomeric matrix protein.

### Use of serum biomarkers to predict OA

Ultimately, we are interested in the information about disease state that measurement of these serum biomarkers can convey. Moreover, because of the wide distribution of predictive covariates, it is plausible that the use of all three systemic biomarkers could suffice to identify which joint-system is affected. To take steps in this direction we sought to determine which covariates, including risk factors (age, height, and weight) and biomarkers, are most useful for predicting the OA burden in each of the joint systems studied. For this purpose we used exhaustive testing of all 2^6^ = 64 linear regression models with interactions among predictors excluded for each of the 2×6 outcomes of interest. The outcome variables used were the percent maximum osteophyte burden and percent maximum joint-space narrowing burden, defined as the sum of the radiographic osteophyte or joint-space narrowing scores, respectively, over all joints within each of the six joint systems, or over all joints in total, divided by the maximum of the relevant sum (maximum sum scores for radiographic features provided in Table S2), and expressed as a percentage. Model selection, as above, was based on the AIC. To determine the proportion of the total variance attributable to the biomarker component of the model, we recomputed the regression with the biomarkers removed. The results of these analyses for total body burden, including Akaike importance weights, are presented in Table 2; complete results for all joint systems are presented in Supplementary Table S3. In all cases, namely individual joint systems and total body burden of disease, the addition of biomarkers to traditional covariates increased the proportion of variance in OA explained. A total of 35–38% of the variance in total body burden of OA (total JSN or total OST) was accounted for by the full model with all three biomarkers, a much smaller proportion of the variance was accounted for in the individual joint systems (Table S3). Total body burden of OST could be optimally predicted with sHA and uCTX2 alone, while total body burden of JSN was best predicted by the use of all three biomarkers.

**Table 2:** Predictors and their coefficients comprising the minimum-AIC models for percentage of maximum osteoarthritis burden.

	Total burden		Akaike importance	
	OST	JSN	OST	JSN
age	0.33 (0.03)	0.52 (0.05)	1.00	1.00
weight	2.12 (1.35)	-	0.82	0.42
height	-	-	0.41	0.28
*s*HA	1.39 (0.37)	1.84 (0.53)	1.00	0.99
*s*COMP	-	−2.70 (0.82)	0.30	0.99
*u*CTX2	2.35 (0.31)	2.04 (0.45)	1.00	1.00
R^2^ no biomarkers	26%	28%		
R^2^ with biomarkers	38%	35%		

AIC  =  Akaike's Information Criterion.

*s*HA  =  serum hyaluronan.

*s*COMP  =  serum cartilage oligomeric matrix protein.

*u*CTX2  =  urinary C-terminal telopeptide of type II collagen normalized to urinary creatinine.

## Discussion

We have suspected for some time that the correlation of systemic biomarkers with disease has been hampered by the inability to fully phenotype the burden of OA in a patient, and in a sense, giving systemic biomarkers a ‘bad name’. The results presented here illustrate the strong correlation of total body burden of disease and several biomarkers, revealed upon adequate patient phenotyping. These results clearly show that all three biomarkers, *s*HA, *s*COMP and *u*CTX2 are quantitative traits of the radiographic feature of OST while *s*COMP is a negative indicator of joint space narrowing affected joint faces in the body. This paper may serve some in the relevant medical communities as an introduction to some novel methods for assessing biomarkers in combination, joint systems in combination, and OA features in combination. For instance, evaluated in combination using multimodel inference with linear mixed-effects regression, it becomes evident that *s*COMP adds little information for predicting total OST burden when *s*HA and *u*CTX2 are available, although *s*COMP is a marker of osteophytes.

There have been several previous studies that used OA-related biomarkers in combination to quantify and characterize the OA process [16], [19], [26], [30], [31], [32]. Four notable examples [16], [26], [31], [32] used principal components analysis (PCA). Meulenbelt *et. al.* identified three components that reflected radiographic OA at different joint sites. The components were comprised of 1) structural markers of cartilage (uCTX2, uTINE, uGlc-Gal-PYD) and bone turnover (uCTX-1 and total sOsteocalcin) associated with hip radiographic OA; 2) a marker of inflammation (serum hsCRP) associated with knee OA, high Western Ontario and McMaster Universities (WOMAC) scores and BMI; and 3) markers of cartilage turnover (sPIIANP, sCOMP) associated with radiographic OA of the hands and lumbar spine as well as age. PCA by Davis *et. al.* has also identified several factors based on biomarkers that correlated with OA features: 1) a factor representing osteophytes that overlapped, as expected, with a factor that correlated with Kellgren Lawrence score; 2) a separate factor that correlated with subchondral bone mineral density; and 3) a factor that correlated with joint space width that overlapped with biomarkers associated with both osteophytes and bone mineral density [16]. In work by Garnero *et al* [31], 10 markers segregated into 5 factors: Factor 1 comprised markers of bone (S-PINP, U-CTX-I) and cartilage (U-CTX2) turnover; factor 2 comprised of SCOMP, S-HA, and S-PIIINP; factor 3 comprised of markers of systemic inflammation S-CRP and S-YKL-40; and factors 4 and 5 comprised of MMP-1 and MMP-3 respectively. Prior work by Otterness *et al* [32] showed that 14 biomarkers segregated into 5 rational groups based on inflammation, bone turnover, cartilage anabolism, cartilage catabolism and transforming growth factor beta.

These studies support our finding that different biomarkers report on different aspects of joint pathology. Specifically, different biomarkers may reflect different molecular pathobiologic mechanisms and different joint contributions to the systemic biomarker concentrations. The interpretation of biomarker levels may be further complicated by the complex biology represented by a biomarker. For instance, decreased levels may reflect reduced matrix degradation, decreased synthesis, or impaired release from the tissue or origin [33]. Osteophyte formation can be considered an anabolic phenomenon and all three biomarkers reported independently on this feature of joint pathology. In contrast, joint space narrowing can be considered a phenomenon of catabolism in excess of anabolism, or a net failure of repair. The negative correlation of COMP and joint space narrowing could represent the failed repair phenomenon (waning of an anabolic epitope) with increased disease severity, or depletion of a catabolic epitope with increased disease severity. In this cohort, neither HA nor CTX2 reported independently on JSN. These findings only came to light with comprehensive phenotyping that demonstrated the utility of total body measures of OA when qualifying a systemic OA-related biomarker for a specific purpose. Moreover, these results also highlight the importance of accounting for both OST and JSN. Failure to account for both is problematic and may lead to spurious conclusions as demonstrated here through comparing and contrasting the results obtained from adjusted versus unadjusted analyses (Figure 2 versus Figure S3, and Figure 3 versus Figure S5).

The biomarker uCTX2 was independent of age while sHA and sCOMP increased with age; uCTX2 was also independent of height and weight, as was sCOMP while sHA increased with weight and decreased with height. The biomarkers increased the variance explained over simple demographic factors for predicting total body burden of disease. The different proportions of variance explained by biomarkers for the different joint systems may be related to genetic variation or to measurement variation, i.e. adequacy of phenotyping. In this regard, it is interesting that biomarkers added most to the prediction of knee OA, for which a standardized radiographic phenotyping procedure was used. This specialized x-ray may more accurately represent the disease. Thus, the biomarkers may not be so marginal but rather, true correlations of systemic biomarkers with disease burden may be higher, and only demonstrable with better gold standards for measuring the disease.

The coefficients of a linear model estimate the average contribution per affected joint to the serum concentration of a biomarker. OST and JSN had very similar coefficients for *s*HA and *uCTX2*, however, there were cases where the difference among OST and JSN were significant and may have real consequences for the use of these biomarkers. One example of the potential complex interrelationship of OST and JSN was demonstrated by the increase of *u*CTX2 with increasing number of CMC joint faces affected by JSN. When the number of joint faces affected by JSN was held constant, however, *u*CTX2 declined with increasing number of CMC joint faces affected by OST. Another point, convincingly demonstrated herein by the large contribution of CMC-OST to both *s*HA and *u*CTX2 concentrations, is that joint size does not necessarily determine the contribution of a joint to the systemic biomarker concentration. One could truly say, based on these data that the CMC “sticks out like a sore thumb”. We do not know why the CMC joint, despite its small size, disproportionally impacted the concentration of all three systemic biomarkers. It is possible that the stage of disease accounted for this phenomenon. For instance, relative to other joints, increasing JSN in the CMC represented increasingly active matrix turnover while increasing CMC osteophyte severity, an anabolic phenomenon, represented waning activity with respect to biomarker production. Another possible explanation relates to relative turnover and clearance of the biomarkers from the CMC joint. Strong evidence exists for differences in joint tissue turnover for different joint systems, best exemplified by differences between knees and ankles [34].

Radiographic features of OST and JSN are interrelated and this correlation can prove to be a potential confounder in attempts to characterize the independent contributors to the systemic level of a biomarker. There are just two examples of OST and JSN features from a single joint system contributing independently to the concentration of a systemic biomarker: MCP on *s*COMP, and CMC on *u*CTX2 (Table 1). In both cases, JSN and OST features had opposing effects on biomarker concentration. This study was limited to women, so these results will need to be validated in other cohorts, including men, selected by other means. Further, because the study participants were selected on the basis of familial hand OA, they may have had greater correlation of OA among joint sites than individuals without so clearly a familial etiology of OA. For this reason, the generalizability of these results would need to be evaluated. Finally, these analyses were limited to the association of a discrete set of biomarkers and structural changes. The field would benefit from similar analyses of a broader range of biomarkers, and qualification studies related to clinical and symptomatic patient-reported outcomes to complement those presented here related to structural endpoints.

## Materials and Methods

### Ethics statement

Written informed consent was obtained from all participants and the study was approved by the institutional review boards of both Duke University and the University of North Carolina at Chapel Hill. The investigation was conducted in accordance with the principles expressed in the Declaration of Helsinki.

### Participants

The participants for these analyses were enrolled between 1999 and 2002 in the GOGO (Genetics of Generalized Osteoarthritis) study and evaluated at two sites (Duke University and the University of North Carolina at Chapel Hill) [6]. A qualifying family consisted of at least 2 siblings with self-reported Western European descent who fulfilled clinical GOGO hand OA criteria: bony enlargement of ≥3 joints distributed bilaterally, including bony enlargement of at least one DIP joint, and no more than 3 swollen MCP joints. Recruitment was independent of clinical symptoms. Once the sibling pair was positively identified as being clinically affected, the nuclear family was invited to participate, together with potential affected or unaffected siblings beyond the required two affecteds. The cohort consisted of 1060 individuals, 840 women and 220 men. Serum and urine biomarker analyses were conducted on the 461 women from this cohort for whom full radiographic data were available. For this study we focused on the 461 women because OA patterns may vary between sexes.

### Phenotypic characterization

Weight and height were measured. Participants underwent radiographic evaluation of both hands, hips, knees and lumbar spine as previously reported [6]. Radiographs were scored for Kellgren Lawrence grade (0–4 scale) [24], and individual radiographic features of OA, osteophyte (OST) and joint space narrowing (JSN), on a 0–3 scale using a standard photographic radiological atlas [35]. All joints, including the lumbar spine, were scored for OST. JSN was scored for DIP, PIP and CMC finger joints, MCP hand joints, knees and hips. The lumbar spine was graded for disc space narrowing which, for purposes of these analyses, was considered JSN. Multiple compartments (or levels in the case of lumbar spine) were graded for these features. The scoring system and possible scores for each joint system are summarized in Table S2 and the number of affected joint systems by KL grade is provided in Table S1. Of the phenotypic data collected, age, and measured weight and height were used in these analyses.

### Biomarker analyses

Blood for sera, and an aliquot of unspun urine, were collected and stored at −80°C for biomarker analyses. Serum samples were analyzed in duplicate for *s*HA using a commercially available kit (Corgenix, Westminster, CO), and *s*COMP using an in-house enzyme-linked immunosorbent assay (ELISA) as previously described [36]. The minimum detectable concentrations of *s*HA and *s*COMP were 10 ng/ml and 120 ng/ml, and intra-and inter-assay coefficients of variation (CVs) were <5% and <7% for *s*HA, and 2.1% and 14.2% for *s*COMP. Urinary CTX2 (Nordic Bioscience Diagnostics CartiLaps®, Herlev, Denmark) was measured per the manufacturer's instructions by competitive ELISA to detect the degradation product, C-terminal telopeptides of type II collagen (*u*CTX2) in urine samples. The concentration of creatinine was measured using a commercially available colorimetric kit (METRA™ QUIDEL Corp., San Diego, CA), and *u*CTX2 values were corrected for urine creatinine concentration (mmol/L). The minimum detectable concentration of *u*CTX2 is reported as 0.20 ng/ml by the manufacturer. Intra-and inter-assay CVs were 5.9% and 9.9%, respectively. There is strong evidence for association with OA at specific joint sites for each of these biomarkers [12], [17], [25], [37], [38], [39] but association with total body burden of disease has not been attempted previously.

### Statistical methods

All statistical analyses were performed using S-Plus 7.0 (Insightful Corp). For each of the three proteins (*s*HA, *s*COMP and *u*CTX2), we fit linear mixed-effects models using family as the random factor. These models allowed us to account for the possibility that there was an inheritable component of constitutive levels of expression for these proteins given that most subjects had one or more relatives in the database. Model estimation was done using Maximum Likelihood (rather than Restricted Maximum Likelihood) to permit comparisons among models using Akaike's Information Criterion (AIC). AIC provides a score that represents the balance between model fit and the number of parameters (model complexity) [29]. Lower values for the AIC indicate more suitable models. Predictor selection was performed using AIC and exhaustive enumeration of all first-order linear models over both mixed-effects models and fixed-effects models (the latter of which exclude family as a predictive covariate). R^2^ values reported for mixed-effects models are computed using Nagelkerke's generalized R^2^ [40]. Model averaging was performed by computing the Akaike weight for each model as 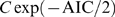 where *C* is a normalizing constant, and summing over all first-order linear models [29]. Adjustments for the risk factors age, height, and weight were made individually for each biomarker by finding the combination of these factors that give the lowest AIC. This procedure provided the “base model” against which all further comparisons were made.

The S-language scripts used to run these analyses are available upon request.

## Supporting Information

Table S1 Classification of participants (N = 461) by radiographic osteoarthritis status.
(0.03 MB DOC)

Table S2 Scoring system for radiographic features of osteoarthritis.
(0.04 MB DOC)

Table S3 Predictors and their coefficients comprising the minimum-AIC models for percentage of maximum osteoarthritis burden.
(0.06 MB DOC)

Figure S1 Age-related prevalence of OA based on radiographic features. Prevalence (total number of cases in the age group, divided by the number of individuals in the age group) of OA of affected joint systems based on any osteophyte (Fig. S1A), or any joint space narrowing (Fig. S1B) by decade of age. The error bars show exact binomial 95% confidence intervals. The numbers of subjects with OA of affected joint systems based on the Kellgren Lawrence grade >1 grade (Fig. S1C) by decade of age. The joint subtypes evaluated included the interphalangeal (IP) finger joints (combination of distal and proximal interphalangeal joints), metacarpophalangeal (MCP or knuckle) hand joints, carpometacarpal (CMC or base of thumb) joint, lumbar spine, hip, and knee joints.
(0.25 MB PDF)

Figure S2 Biomarkers and number of affected joint systems based on traditional Kellgren Lawrence (KL) scoring. Mean log sHA (top), log sCOMP (middle), and log uCTX2 (bottom), by number of affected joint systems with OA based on Kellgren Lawrence (KL) grade >1. These models were fit with the following risk factors: age, log height, log weight for sHA; age for sCOMP; and for uCTX2 no other covariate was used. The respective R2 values, giving the proportion of the among-KL grade variation explained by the linear regression are shown. Accompanying p-values are 3.6×10−7, 0.024, and 3.6×10−5.
(0.66 MB TIF)

Figure S3 Biomarkers and total burden of OA based on radiographic features (unadjusted). Point estimates are plotted for mean serum log sHA (top), log sCOMP (middle), and log uCTX2 (bottom) by number of joint faces affected by any osteophyte (grade >0, left panels), and number of joint faces OA affected by any joint space narrowing (grade >0, right panels). These unadjusted data are a companion to the adjusted data shown in Figure 2.
(3.00 MB TIF)

Figure S4 Examples of biomarker concentrations and radiographic features of OA in specific joint systems (adjusted). Point estimates, 95% confidence intervals, and mixed-model regression lines for mean serum log sHA (top), log sCOMP (middle), and log uCTX2 (bottom) by number of joint faces affected by any osteophyte (grade >0, left panels), and number of joint faces affected by any joint space narrowing (grade >0, right panels). Each panel represents an analysis that starts with the optimal mixed-effects model for each biomarker as developed in the text, and supplements it with the predictor of interest if it is not already in the optimal model. The line in each plot represents the prediction under the supplemented mixed-effects model. R2 values are indicated giving the among-radiography class explained variation. Corresponding p-values are (left to right by row): 1.9×10−4, 0.039, 0.11, 3.3×10−4, 1.3×10−3, 0.18.
(1.98 MB TIF)

Figure S5 Linear coefficients of biomarker associations with radiographic features of OA (unadjusted). In this example, features are fit independently, so that correlations among features are not accounted for; the relationships between features and biomarkers in this analysis are complicated by these correlations (compare with Figure 3). Linear coefficients for each joint group and radiographic feature are shown.
(1.71 MB TIF)

Figure S6 Example of complex interaction of OST and JSN on a biomarker concentration. Mean log uCTX2 concentrations varied positively with JSN but negatively with OST. For the depicted component of the mixed-effects model, R2 = 0.977, p = 5.4×10−4.
(1.05 MB TIF)
