# Supplementary material for: First Qualification Study of Serum Biomarkers as Indicators of Total Body Burden of Osteoarthritis
Source: PLoS One. 2010 Mar 17;5(3):e9739. doi: 10.1371/journal.pone.0009739 (PMC2840035; doi:10.1371/journal.pone.0009739)
Supplement: Table S1 — Classification of participants (N = 461) by radiographic osteoarthritis status. (0.03 MB DOC) [file pone.0009739.s001.doc]

**Table S1.** Classification of participants (N=461) by radiographic osteoarthritis status.

| **Joint System** | **N affected based on**  **KL grade > 1** |
| --- | --- |
|
| Hand - IP | 419 |
| Hand - MCP | 238 |
| Hand - CMC | 246 |
| Lumbar Spine | 248 |
| Hip | 175 |
| Knee | 169 |
| **Number of any combination of involved systems (**IP, MCP, CMC, Lumbar Spine, Hip, Knee**)*** | **N affected by total number of systems** |
| 0 | 13 |
| 1 | 58 |
| 2 | 84 |
| 3 | 103 |
| 4 | 92 |
| 5 | 74 |
| 6 | 37 |

KL = Kellgren Lawrence traditional grading system based on a combination of osteophyte and joint space narrowing features.

IP = interphalangeal (distal and proximal) finger joints

MCP = metacarpophalangeal (knuckle) hand joints

CMC = first carpometacarpal phalangeal (base of thumb) joint

*Individuals with no affected systems represented additional female siblings invited to participate.
