# Supplementary material for: First Qualification Study of Serum Biomarkers as Indicators of Total Body Burden of Osteoarthritis
Source: PLoS One. 2010 Mar 17;5(3):e9739. doi: 10.1371/journal.pone.0009739 (PMC2840035; doi:10.1371/journal.pone.0009739)
Supplement: Table S2 — Scoring system for radiographic features of osteoarthritis. (0.04 MB DOC) [file pone.0009739.s002.doc]

**Table S2. Scoring system for radiographic features of osteoarthritis.**

| Joint Site | Range of OST scores for a single joint | Range of JSN scores for a single joint | Number of joints per system | Range of OST scores for joint group | Range of JSN score for joint group |
| --- | --- | --- | --- | --- | --- |
| Hand - DIP | 0-3 | 0-3 | 8 | 0-24 | 0-24 |
| Hand - PIP | 0-3 | 0-3 | 10 | 0-30 | 0-30 |
| Hand - MCP | 0-3 | 0-3 | 10 | 0-30 | 0-30 |
| Hand - CMC | 0-3 | 0-3 | 2 | 0-6 | 0-6 |
| Knee | 0-24 | 0-12 | 2 | 0-48 | 0-24 |
| Hip | 0-12 | 0-9 | 2 | 0-24 | 0-18 |
| Lumbar Spine | 0-6 | 0-3 | 5 | 0-30 | 0-15 |

Knee included scoring of the medial and lateral tibiofemoral, and patellofemoral compartments.

Hip included scoring of the superior, axial and medial compartments.

Lumbar Spine included scoring of the 5 lumbar levels.

The thumb interphalangeal was considered a proximal interphalangeal (PIP) joint for purposes of these analyses.

Osteophytes were summed over the four corners of the knee, and hip, respectively, and over the top and bottom vertebral faces for the spine.

OST = osteophyte

JSN = joint space narrowing
