# Supplementary material for: First Qualification Study of Serum Biomarkers as Indicators of Total Body Burden of Osteoarthritis
Source: PLoS One. 2010 Mar 17;5(3):e9739. doi: 10.1371/journal.pone.0009739 (PMC2840035; doi:10.1371/journal.pone.0009739)
Supplement: Table S3 — Predictors and their coefficients comprising the minimum-AIC models for percentage of maximum osteoarthritis burden. (0.06 MB DOC) [file pone.0009739.s003.doc]

**Table S3.** Predictors and their coefficients comprising the minimum-AIC models for percentage of maximum osteoarthritis burden.

|  | IP | | MCP | | CMC | | Lumbar Spine | | Hip | | Knee | | Total body | |
| --- | --- | --- | --- | --- | --- | --- | --- | --- | --- | --- | --- | --- | --- | --- |
|  | OST | JSN | OST | JSN | OST | JSN | OST | JSN | OST | JSN | OST | JSN | OST | JSN |
| age | 1.67  (0.18) | 1.86  (0.16) | 0.27  (0.04) | 0.4  (0.08) | 0.75  (0.14) | 1.05  (0.13) | 0.25  (0.06) | 0.35  (0.06) | 0.06  (0.02) | 0.13  (0.03) | 0.15  (0.05) | 0.23  (0.05) | 0.33 (0.03) | 0.52 (0.05) |
| weight | -10.95  (7.36) |  |  | -9.8  (3.64) |  |  | 14.71  (2.91) |  |  |  | 11.56  (2.11) | 7.24  (2.05) | 2.12 (1.35) |  |
| height |  |  |  |  | 53.76  (29.62) |  |  |  |  |  |  |  |  |  |
| *s*HA | 5.57  (2.04) | 5.52  (1.98) |  |  | 6.71  (1.57) | 3.33  (1.55) |  |  |  | 0.61  (0.36) | 2.52  (0.57) | 2.77  (0.56) | 1.39 (0.37) | 1.84 (0.53) |
| *s*COMP | -8.92  (3.13) | -11.22  (3.06) | 2.41  (0.92) |  |  | -7.97  (2.39) | 2.81  (1.22) |  |  |  |  |  |  | -2.70 (0.82) |
| *u*CTX2 | 8.85  (1.71) | 4.31  (1.66) | 2.44  (0.5) | 4.6  (0.84) | 2.92  (1.34) | -4.82  (1.3) | 1.92  (0.67) | 1.85  (0.64) | 1.04  (0.3) |  | 3.07  (0.49) | 2.62  (0.47) | 2.35 (0.31) | 2.04 (0.45) |
| R^2^ no biomarkers | 23.5% | 25.9% | 7.6% | 8.5% | 10.1% | 13.8% | 8.2% | 7.2% | 1.4% | 5.3% | 11.9% | 12.2% | 26.0% | 28.0% |
| R^2^ biomarkers | 30.3% | 30.0% | 13.7% | 14.1% | 15.5% | 18.4% | 11.0% | 8.8% | 3.8% | 5.9% | 23.6% | 23.3% | 37.7% | 34.6% |

AIC = Akaike’s Information Criterion

IP = interphalangeal (distal and proximal) finger joints

MCP = metacarpophalangeal (knuckle) hand joints

CMC = first carpometacarpal phalangeal (base of thumb) joint

*s*HA = serum hyaluronan

*s*COMP = serum cartilage oligomeric matrix protein

*u*CTX2 = urinary C-terminal telopeptide of type II collagen normalized to urinary creatinine
