# Supplementary figures and images for: First Qualification Study of Serum Biomarkers as Indicators of Total Body Burden of Osteoarthritis
Source: PLoS One. 2010 Mar 17;5(3):e9739. doi: 10.1371/journal.pone.0009739 (PMC2840035; doi:10.1371/journal.pone.0009739)

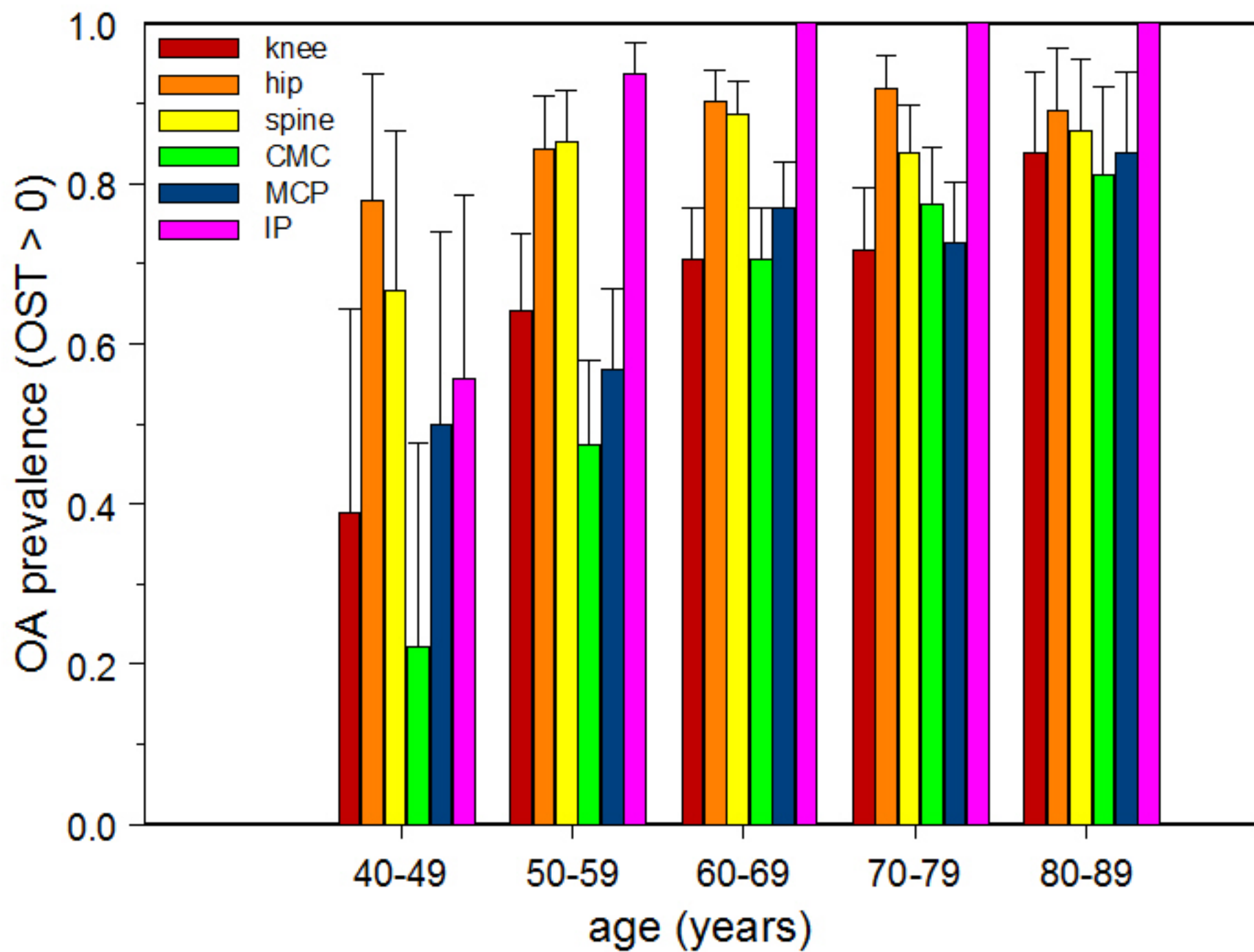

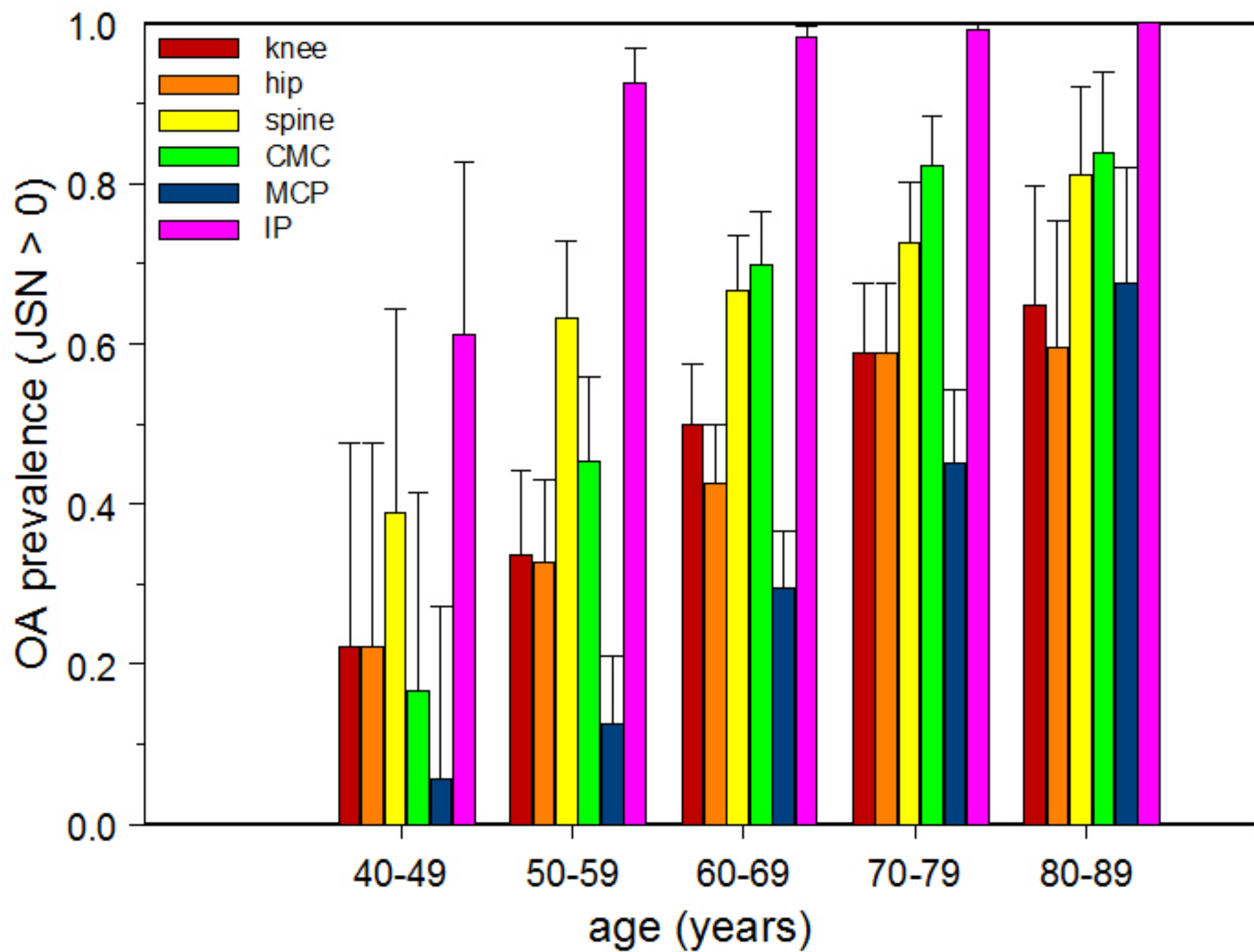

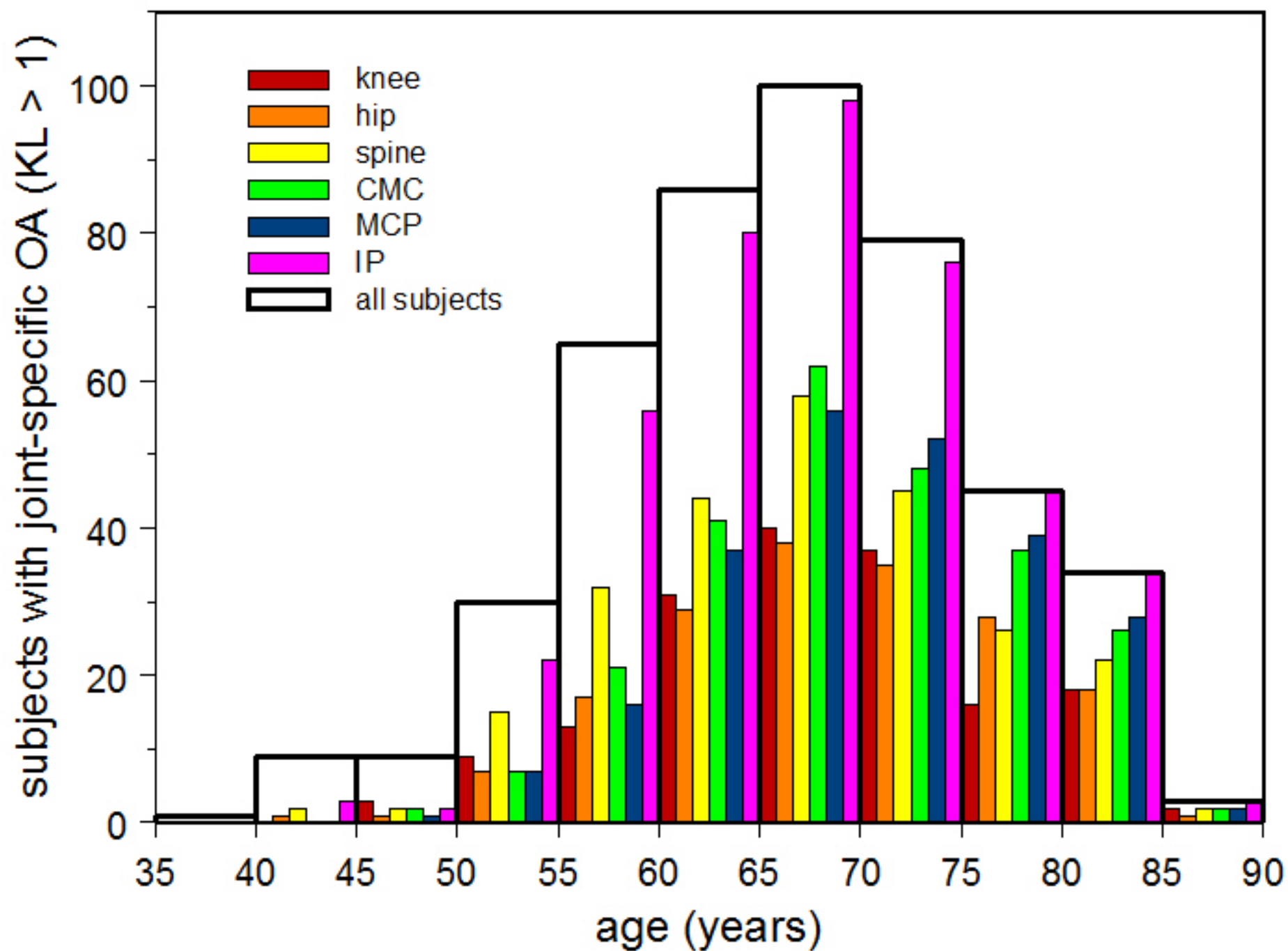

Supplement: Figure S1 — Age-related prevalence of OA based on radiographic features. Prevalence (total number of cases in the age group, divided by the number of individuals in the age group) of OA of affected joint systems based on any osteophyte (Fig. S1A), or any joint space narrowing (Fig. S1B) by decade of age. The error bars show exact binomial 95% confidence intervals. The numbers of subjects with OA of affected joint systems based on the Kellgren Lawrence grade >1 grade (Fig. S1C) by decade of age. The joint subtypes evaluated included the interphalangeal (IP) finger joints (combination of distal and proximal interphalangeal joints), metacarpophalangeal (MCP or knuckle) hand joints, carpometacarpal (CMC or base of thumb) joint, lumbar spine, hip, and knee joints. (0.25 MB PDF) [file pone.0009739.s004.pdf]

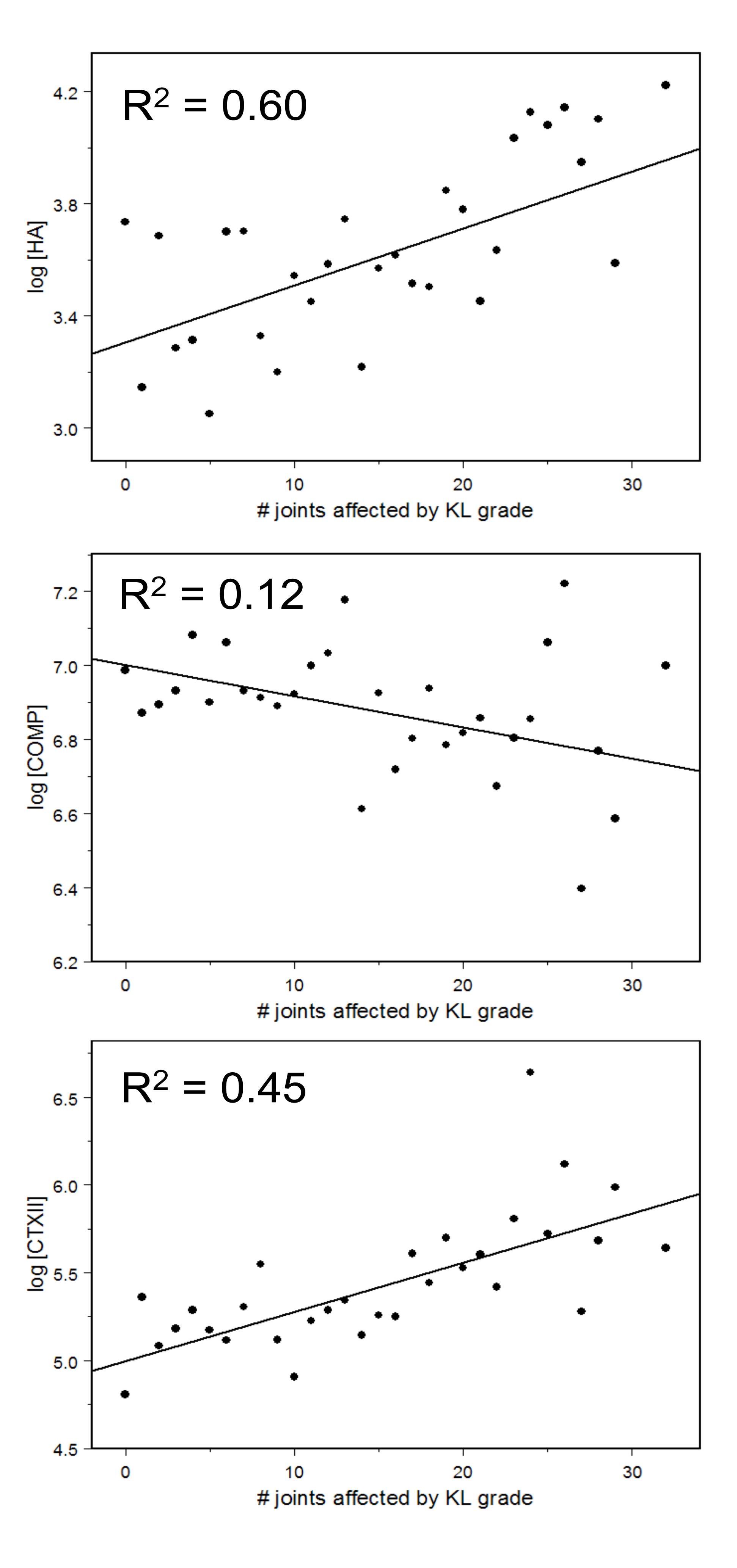

Supplement: Figure S2 — Biomarkers and number of affected joint systems based on traditional Kellgren Lawrence (KL) scoring. Mean log sHA (top), log sCOMP (middle), and log uCTX2 (bottom), by number of affected joint systems with OA based on Kellgren Lawrence (KL) grade >1. These models were fit with the following risk factors: age, log height, log weight for sHA; age for sCOMP; and for uCTX2 no other covariate was used. The respective R2 values, giving the proportion of the among-KL grade variation explained by the linear regression are shown. Accompanying p-values are 3.6×10^−7^, 0.024, and 3.6×10^−5^. (0.66 MB TIF) [file pone.0009739.s005.tif]

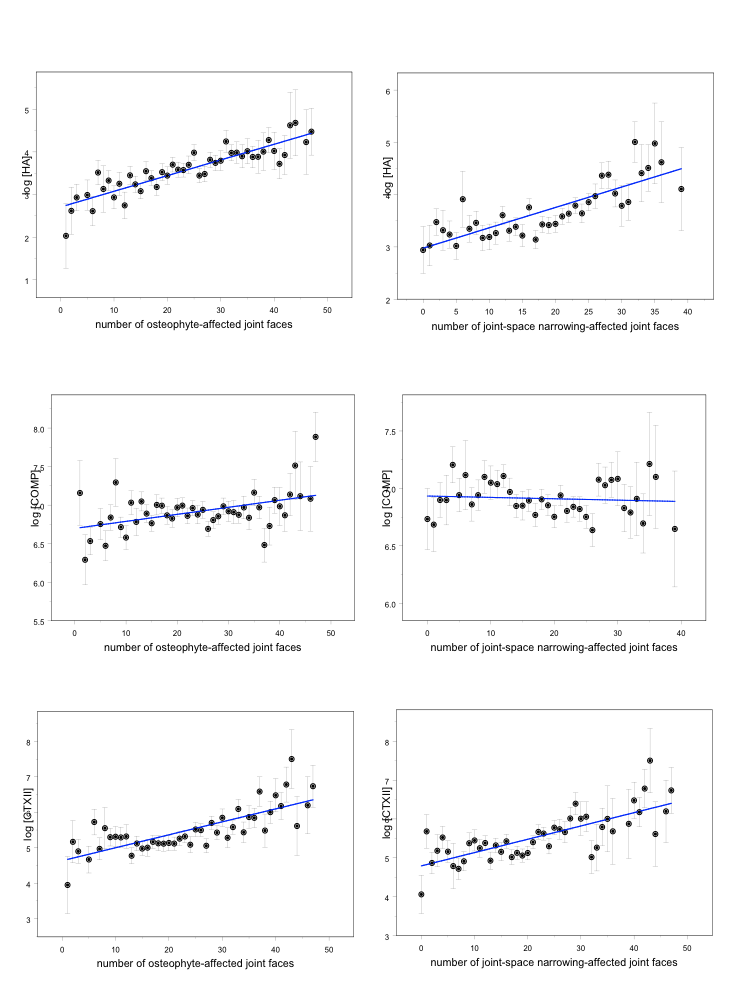

Supplement: Figure S3 — Biomarkers and total burden of OA based on radiographic features (unadjusted). Point estimates are plotted for mean serum log sHA (top), log sCOMP (middle), and log uCTX2 (bottom) by number of joint faces affected by any osteophyte (grade >0, left panels), and number of joint faces OA affected by any joint space narrowing (grade >0, right panels). These unadjusted data are a companion to the adjusted data shown in Figure 2. (3.00 MB TIF) [file pone.0009739.s006.tif]

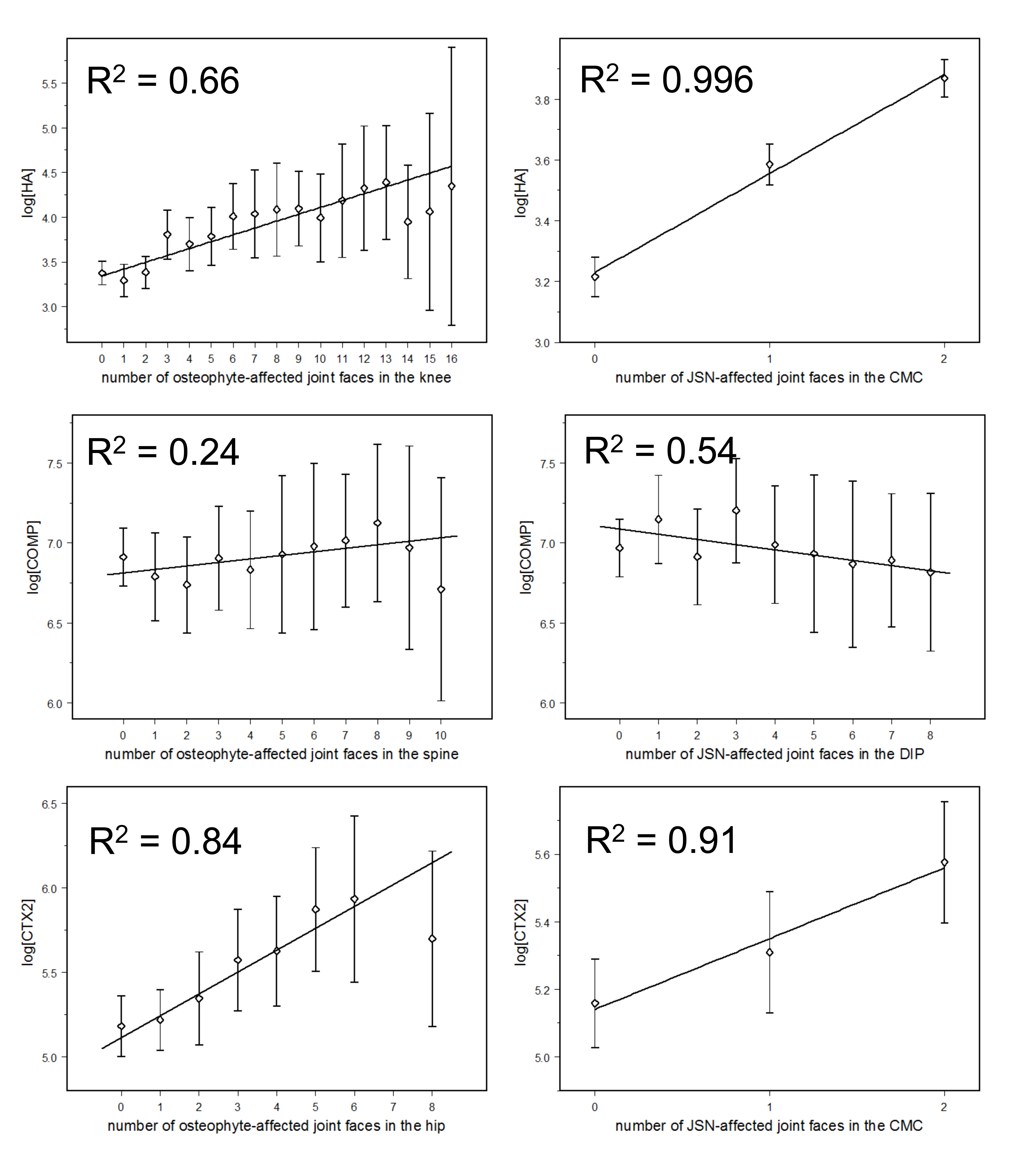

Supplement: Figure S4 — Examples of biomarker concentrations and radiographic features of OA in specific joint systems (adjusted). Point estimates, 95% confidence intervals, and mixed-model regression lines for mean serum log sHA (top), log sCOMP (middle), and log uCTX2 (bottom) by number of joint faces affected by any osteophyte (grade >0, left panels), and number of joint faces affected by any joint space narrowing (grade >0, right panels). Each panel represents an analysis that starts with the optimal mixed-effects model for each biomarker as developed in the text, and supplements it with the predictor of interest if it is not already in the optimal model. The line in each plot represents the prediction under the supplemented mixed-effects model. R2 values are indicated giving the among-radiography class explained variation. Corresponding p-values are (left to right by row): 1.9×10^−4^, 0.039, 0.11, 3.3×10^−4^, 1.3×10^−3^, 0.18. (1.98 MB TIF) [file pone.0009739.s007.tif]

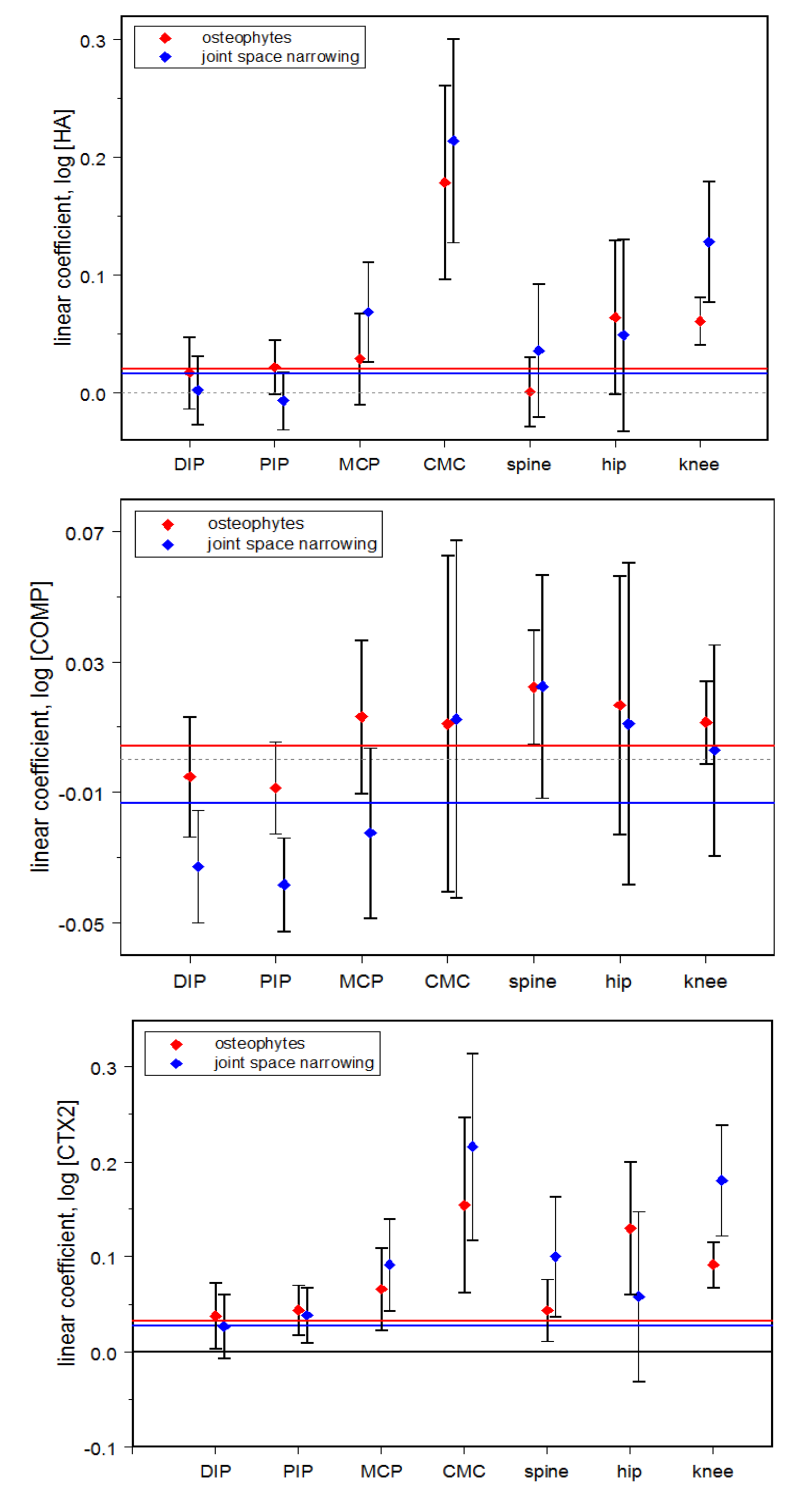

Supplement: Figure S5 — Linear coefficients of biomarker associations with radiographic features of OA (unadjusted). In this example, features are fit independently, so that correlations among features are not accounted for; the relationships between features and biomarkers in this analysis are complicated by these correlations (compare with Figure 3). Linear coefficients for each joint group and radiographic feature are shown. (1.71 MB TIF) [file pone.0009739.s008.tif]

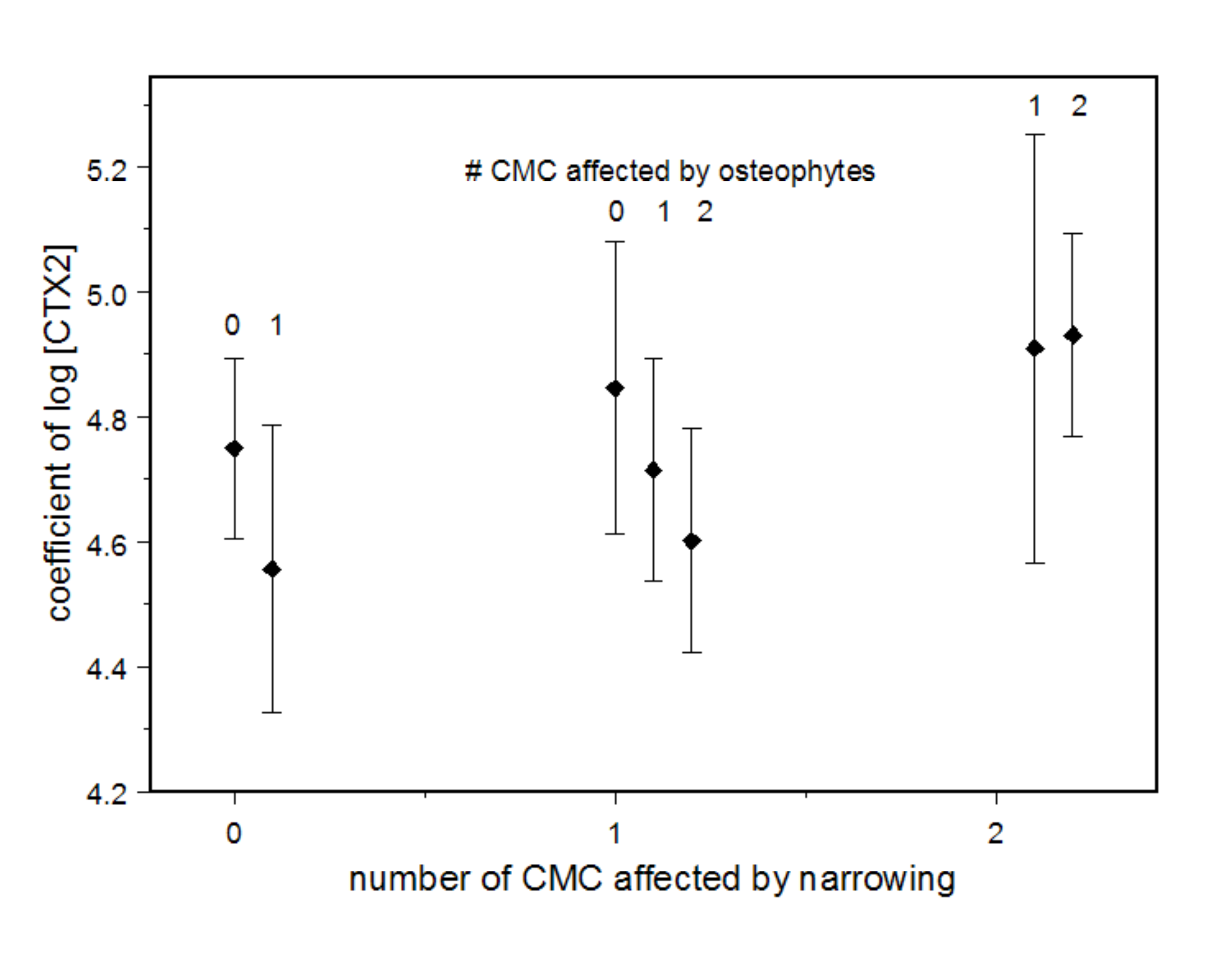

Supplement: Figure S6 — Example of complex interaction of OST and JSN on a biomarker concentration. Mean log uCTX2 concentrations varied positively with JSN but negatively with OST. For the depicted component of the mixed-effects model, R2 = 0.977, p = 5.4×10^−4^. (1.05 MB TIF) [file pone.0009739.s009.tif]
